# Bacterial protease uses distinct thermodynamic signatures for substrate recognition

**DOI:** 10.1038/s41598-017-03220-y

**Published:** 2017-06-06

**Authors:** Gustavo Arruda Bezerra, Yuko Ohara-Nemoto, Irina Cornaciu, Sofiya Fedosyuk, Guillaume Hoffmann, Adam Round, José A. Márquez, Takayuki K. Nemoto, Kristina Djinović-Carugo

**Affiliations:** 1 Department of Structural & Computational Biology, Max F. Perutz Laboratories, University of Vienna, Vienna Biocenter, Vienna Biocenter Campus 5, A-1030 Vienna, Austria; 2 Department of Oral Molecular Biology, Course of Medical and Dental Sciences, Nagasaki University Graduate School of Biomedical Sciences, Nagasaki, 852-8588 Japan; 3 European Molecular Biology Laboratory, Grenoble Outstation, 71 avenue des Martyrs, CS 90181, 38042 Grenoble, France; 4 Max F. Perutz Laboratories, Medical University of Vienna, Vienna Biocenter, Dr. Bohr-Gasse 9/3, A-1030 Vienna, Austria; 5 Department of Biochemistry, Faculty of Chemistry and Chemical Technology, University of Ljubljana, Večna pot 113, SI-1000 Ljubljana, Slovenia; 6 Max F. Perutz Laboratories, Medical University of Vienna, Vienna Biocenter, Dr. Bohr-Gasse 9/3, A-1030 Vienna, Austria; 7 European XFEL GmbH, Notkestraße 85, 22607 Hamburg, Germany

## Abstract

*Porphyromonas gingivalis* and *Porphyromonas endodontalis* are important bacteria related to periodontitis, the most common chronic inflammatory disease in humans worldwide. Its comorbidity with systemic diseases, such as type 2 diabetes, oral cancers and cardiovascular diseases, continues to generate considerable interest. Surprisingly, these two microorganisms do not ferment carbohydrates; rather they use proteinaceous substrates as carbon and energy sources. However, the underlying biochemical mechanisms of their energy metabolism remain unknown. Here, we show that dipeptidyl peptidase 11 (DPP11), a central metabolic enzyme in these bacteria, undergoes a conformational change upon peptide binding to distinguish substrates from end products. It binds substrates through an entropy-driven process and end products in an enthalpy-driven fashion. We show that increase in protein conformational entropy is the main-driving force for substrate binding via the unfolding of specific regions of the enzyme (“entropy reservoirs”). The relationship between our structural and thermodynamics data yields a distinct model for protein-protein interactions where protein conformational entropy modulates the binding free-energy. Further, our findings provide a framework for the structure-based design of specific DPP11 inhibitors.

## Introduction

Periodontitis is the most common chronic inflammatory disease of humans worldwide, affecting nearly half of adults in the United Kingdom and the United States of America^1, 2^. The condition is characterized by destruction of the connective tissue and alveolar bone surrounding the teeth and has many negative impacts in life quality^3^, for instance, loss of permanent tooth. *Porphyromonas gingivalis* ^4^ is the major causative agent in periodontitis and *Porphyromonas endodontalis* ^5^ is another abundant bacterium in periodontal sites. Considerable attention has been drawn to these organisms due to recent reports associating periodontitis to systemic diseases^6^ like type II diabetes mellitus^7^, rheumatoid arthritis^8^, oral cancers^9, 10^, cardiovascular diseases^11^, Alzheimer *et al*.^12^ and respiratory diseases^13^. In particular, *P. gingivalis* is a model pathogen for investigating microbial subversion in periodontal host immune response, which causes adverse impacts in systemic health^14^.

Both *Porphyromonas* species are Gram-negative black-pigmented anaerobes that do not ferment carbohydrates; instead, they use proteinaceous substrates as carbon and energy source^15^. Proteases with different specificities reduce these extracellular proteins into di- and tri-peptides^16^, which are further degraded via specific pathways, producing short-chain fatty acids, ammonia, acetate, propionate and butyrate^17^. Together with other *P. gingivalis* elements such as the recently characterized pili^18^, these metabolic end products are also virulence factors causing host tissue damage^19^. In *P. gingivalis*, extracellular proteins are initially degraded to oligopeptides by potent cysteine endopeptidases, i.e., gingipains R (Rgp, Arg-specific) and K (Kgp, Lys-specific)^20^, mainly localized in the outer membrane. Sequentially, the periplasmic enzymes, four dipeptidyl peptidases (DPPs), (i.e. DPP4, DPP5, DPP7 and DPP11), prolyl tripeptidyl peptidase-A and acylpeptidyl oligopeptidase convert the oligopeptides to di- and tri-peptides^16^, which are then incorporated via oligopeptide transporters^21^. These enzymes’ different specificities and their concerted actions secure proper nutrient source and are essential for the bacteria metabolism. However, metabolic regulation for amino acid degradation is not well understood. Furthermore, these dipeptidases are widely distributed in the bacterial kingdom, including the two major phyla *Bacteroidetes* and *Proteobacteria* ^22^, thus it is of ample relevance to elucidate their mechanism of action.

In *P. gingivalis*, the most utilized peptides contain Asp/Glu^23^ and are degraded by dipeptidyl peptidase 11 (DPP11), rendering it a central metabolic role in this microorganism^24^. The metabolism of glutamate- and aspartate-containing peptides generates cytotoxic products^23, 25^, such as ammonia and butyrate, which may have a role in this bacterium to adversely impact systemic health. DPP11 is a dimeric 162 kDa (Supplementary Fig. S1) periplasmic serine protease (catalytic triad S652, D226 and H85) recently discovered in *P. endodontalis* and later identified in *P. gingivalis* by homology search^24^ (they share close to 58% identity). Due to its specificity for Asp/Glu in the P1 position (second amino acid from the peptide N-terminus), DPP11 discovery is in line with the observation that aspartate and glutamate are the most intensively consumed amino acids in *P. gingivalis* ^23^. Indeed, *P. gingivalis dpp11*-knock-out strain shows growth impairment^24^, suggesting its critical role in the bacterium energy metabolism. Its absence in mammals^26^ strengthens the enzyme’s potential as an attractive drug target. In this way, we aimed at elucidating the structural basis of peptide recognition by DPP11 in order to establish its mechanism of action.

We determined the structures for the inactive constructs PgDPP11_22-720_ S655A, PeDPP11_22-717_ S652A and its complexes with the dipeptides Arg-Asp and Arg-Glu, as well as the substrate Leu-Asp-Val-Trp, at 2.4, 2.85, 2.2, 2.1 and 2.6 Å resolution, referred to as PgDPP11, PeDPP11, PeDPP11:RD, PeDPP11:RE and PeDPP11:LDVWs, respectively (Table 1). DPP11 crystal structures in complex with peptides disclose a significant domain motion upon ligand binding and allow the elucidation of the enzyme’s specificity and selectivity. The distinct conformational states reported here offer opportunities for the rational development of drugs and molecular tools for DPP11 studies, which are not possible to be fully exploited in the unbound form of the enzyme. Microcalorimetric analyse reveal a dual thermodynamic signature where DPP11 binds substrates through an endothermic/entropy-driven process, and end products in an exothermic/enthalpy-driven fashion. We propose that increase in protein conformational entropy is the main-driving force for substrate recognition and that enzyme plasticity favours substrate promiscuity.

**Table 1:** Data collection and refinement statistics.

	PgDPP11	PeDPP11	PeDPP11:RD	PeDPP11:RE
**Data collection**				
X-ray source	BM14/ESRF	ID30A-1/ESRF	ID29/ESRF	ID29/ESRF
Space group	*P* *2*_*1*_ *2*_*1*_ *2*_*1*_	*P* *2*_*1*_ *2*_*1*_ *2*_*1*_	*P* *2*_*1*_ *2*_*1*_ *2*_*1*_	*P* *2*_*1*_ *2*_*1*_ *2*_*1*_
**Cell dimensions**				
*a*, *b*, *c* (Å)	103.18, 117.21, 148.35	76.75, 91.83, 229.91	111.81, 114.40, 147.82	111.44, 112.53, 148.26
Resolution (Å)	47.22–2.20 (2.32–2.20)	48.72–2.85 (3.00–2.85)	48.15–2.20 (2.32–2.20)	49.42–2.10 (2.21–2.10)
*R*_pim_ (%)	4.2 (57.9)	9.4 (46.8)	3.1 (37.9)	4.7 (38.3)
*R*_merge_ (%)	9.1 (122.4)	22.6 (111.8)	6.1 (74.4)	8.4 (69.6)
CC1/2 (%)	99.8 (96.8)	98.9 (53.5)	99.9 (68.5)	99.6 (69.7)
*I* / σ(*I)*	12.3 (0.9)	7.5 (1.7)	14.9 (2.0)	8.8 (2.0)
Completeness (%)	99.7 (99.4)	100 (100)	99.6 (99.1)	99.0 (99.8)
Redundancy	5.6 (5.4)	6.6 (6.6)	4.7 (4.6)	3.9 (4.0)
**Refinement**				
Resolution (Å)	47.22–2.40	46.7–2.85	45.24–2.20	47.47–2.10
No. reflections	70637	38796	9612	107843
*R*_work_ / *R*_free_ (%)	20.7/25.9	24.0/27.4	18.1/22.6	18.9/22.9
No. atoms	11516	10832	11235	11093
Protein	11132	10773	10769	10549
Ligand/ion	23	3	40/5	42/2
Water	361	56	421	500
*B*–factors (A^2^)				
Protein	55.97	46.9	65.3	56.0/
Ligand/ion	72.93	20.78	47.6/62.6	44.6/51.326
Water	51.58	16.6	53.2	47.4
R.m.s. deviations				
Bond lengths (Å)	0.002	0.003	0.008	0.008
Bond angles (°)	0.680	0.763	1.141	1.094
Ramachandran analysis				
Favoured (%)	96	93.5	95.5	95.8
Allowed (%)	3.5	5.8	3.8	3.7
Outliers (%)	0.5	0.6	0.7	0.5
	**PeDPP11:LDVW**	**PeDPP11:altconf**	**FpDPP11:RD**	
**Data collection**				
X–ray source	ID30A–1/ESRF	ID30A–1/ESRF	ID23–1/ESRF	
Space group	*C*2	*C*2	*P*2_1_	
Cell dimensions				
*a*, *b*, *c* (Å)	87.78, 113.33, 111.22	88.02, 103.99, 111.39	126.05, 70.68, 191.59	
	β = 106.2°	β = 104.9º	β = 97.3º	
Resolution (Å)	47.40–2.60 (2.74–2.60)	46.82–2.50 (2.64–2.50)	47.61-2.10 (2.21–2.10)	
*R*_pim_ (%)	8.2 (75.4)	6.3 (43.8)	3.6 (43.2)	
*R*_merge_ (%)	13.0 (121.1)	9.6 (67.4)	5.5 (66.6)	
CC1/2 (%)	99.2 (41.2)	99.5 (73.4)	99.9 (77.5)	
*I* / σ(*I)*	7.5 (1.0)	9.2 (1.5)	12.1 (1.7)	
Completeness (%)	99.2 (95.9)	99.0 (99.6)	98.9 (99.6)	
Redundancy	3.4 (3.5)	3.1 (3.0)	3.2 (3.2)	
**Refinement**				
Resolution (Å)	43.58–2.60	46.85–2.50	46.83–2.10	
No. reflections	32005	33283	193404	
*R*_work_ / *R*_free_ (%)	21.7/24.7	19.9/24.0	19.6/24.4	
No. atoms	5490	5393	22036	
Protein	5399	5240	21499	
Ligand/ion	23/16	3	38/4	
Water	52	150	495	
*B*–factors (A^2^)				
Protein	51.98	48.5	65.5	
Ligand/ion	43.29/90.86	58.5	70.3/58.7	
Water	43.04	41.7	50.3	
R.m.s. deviations				
Bond lengths (Å)	0.014	0.003	0.009	
Bond angles (°)	1.119	0.705	1.149	
Ramachandran analysis				
Favoured (%)	94	95.1	94.7	
Allowed (%)	6	4.1	4.8	
Outliers (%)	0	0.8	0.5	

## Results and Discussion

**Figure 1:**
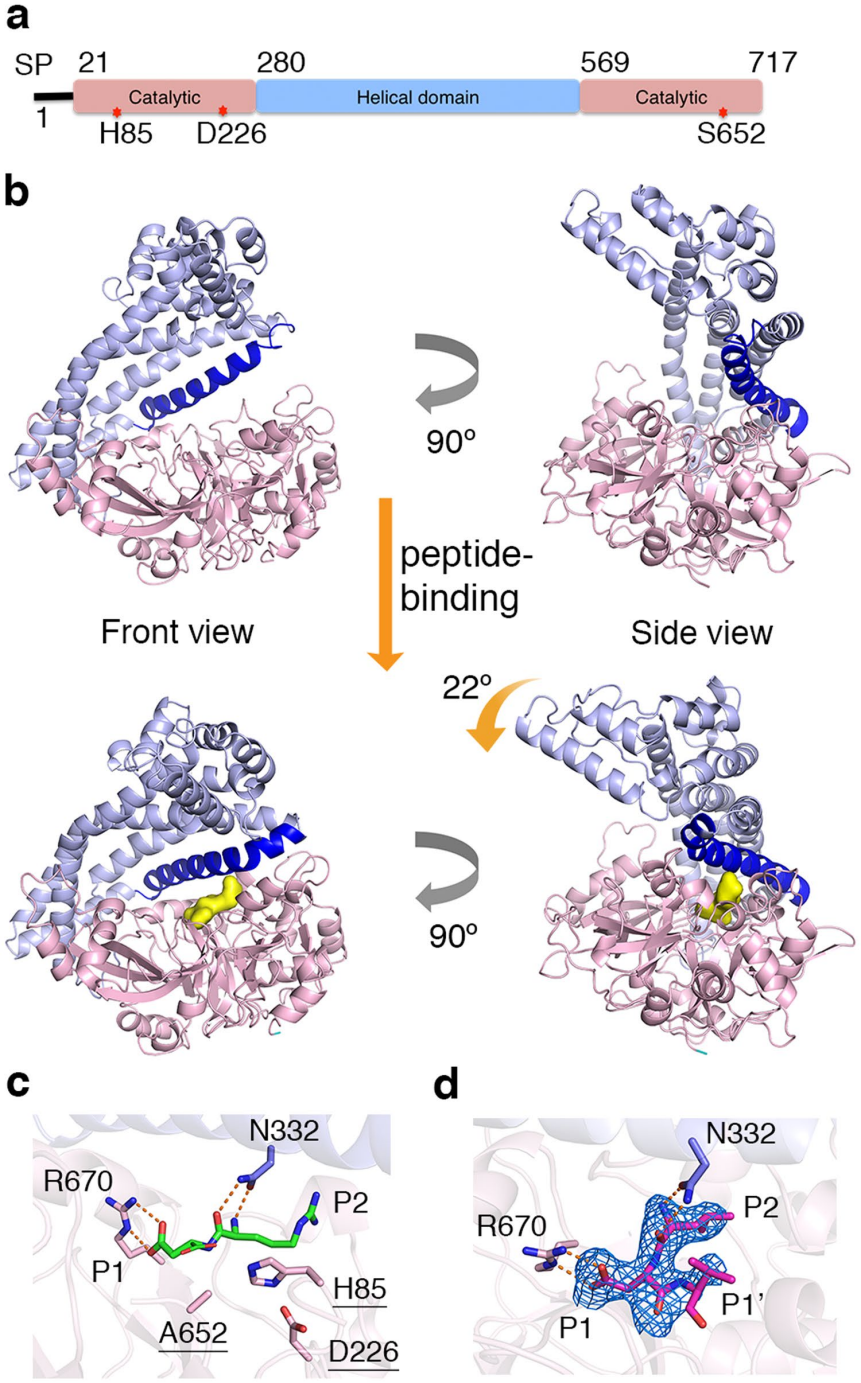
Structure of *Porphyromonas endodontalis* DPP11. **(a)** Domain architecture of PeDPP11. SP is signal peptide. The locations of catalytic triad amino acids are indicated by “red stars”. **(b)** Ribbon representation of PeDPP11 structure. Domains are coloured as in item **(a)** and helix α14 is shown in dark blue. Upper panel shows two perpendicular views of unbound PeDPP11. Lower panel shows two perpendicular views of PeDPP11 as in complex with peptides (binding pocket shown as yellow surface). **(c)** Active site of PeDPP11:RD (peptide RD shown in green). Catalytic triad is underlined. Note that S652 is mutated to alanine. **(d)** Active site of PeDPP11:LDVW (peptide LDVW shown in magenta), peptide omit map contoured at 3σ, shown in blue.

As previously reported^27^, the overall fold of DPP11 comprises a bilobal architecture (Fig. 1a,b). The upper helical domain dictates the specificity of the enzyme and caps the catalytic domain, which has a typical chymotrypsin double β-barrel fold^28^. PeDPP11 and PgDPP11 superposition yielded a root mean square deviation (r.m.s.d.) of 1.4 Å for 629 out of 685 superimposed Cα-atoms. A notable difference between the unbound PeDPP11 and its complexes with peptides is the conformational change bringing the helical and catalytic domains closer (Fig. 1b). This movement yields an approximate rotation of 22° of one domain relative to the other with a negligible translational component^29, 30^. Notably, the helical domain undergoes larger structural changes reflected in higher r.m.s.d. and B-factor values, when compared to the catalytic domain, which behaves as a rigid body (Supplementary Table S1a,b).

**Figure 2:**
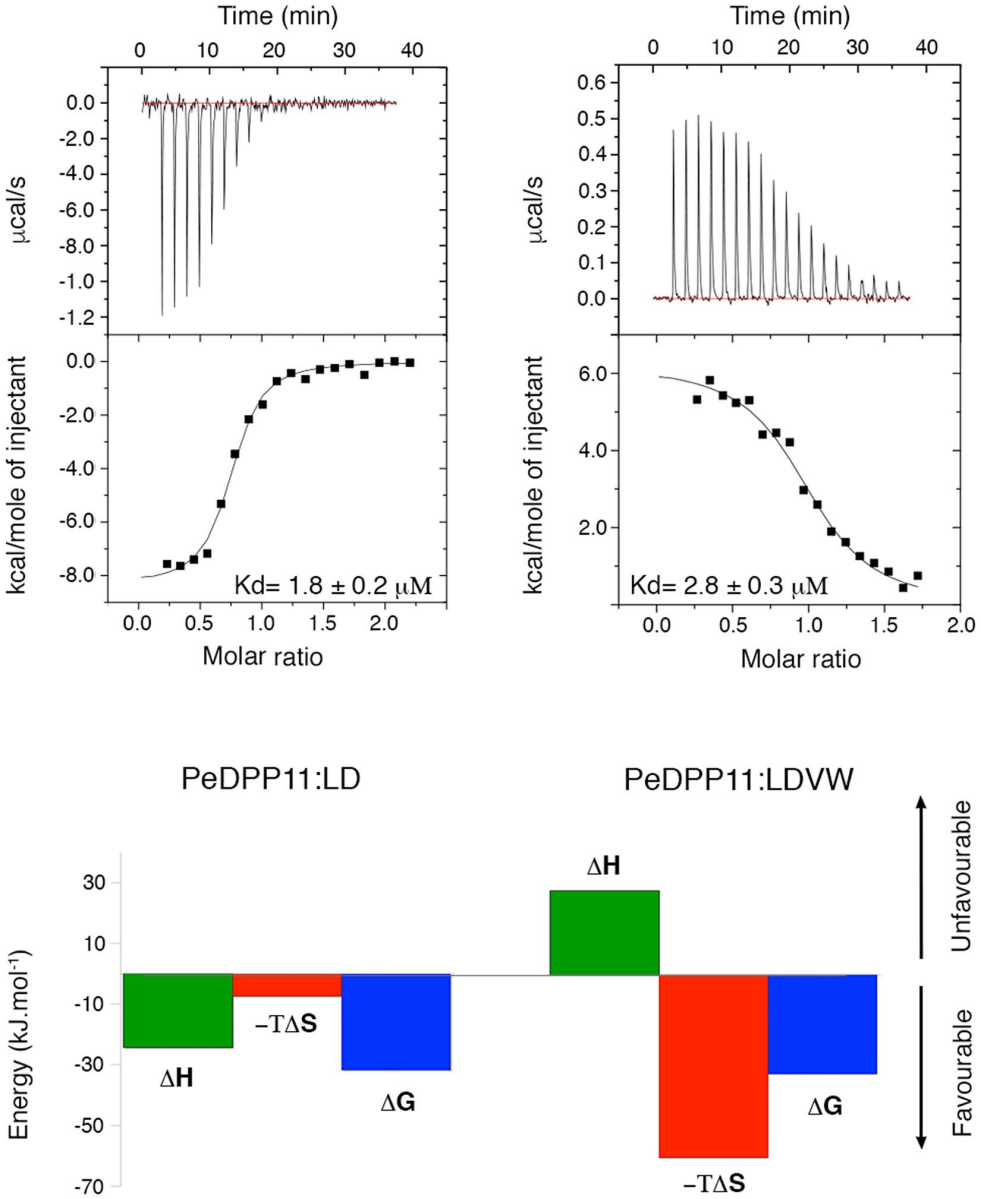
Microcalorimetric analysis. Isothermal titration calorimetry experiments performed by titrating LD (left panel) and LDVW (right panel) into PeDPP11. Upper panel shows time-dependent deflection of heat for each injection (top). Integrated calorimetric data for the respective interactions (bottom). The continuous curve represents the best fit using a one-site binding model. Lower panel shows the graphical representation of thermodynamics parameters.

The active site of DPP11 lays in a wide cleft running through the middle of the protein between the catalytic and helical domains, which contributes to the formation of the substrate binding subsites (Supplementary Table S2a–c). The bound-peptide is anchored at its N-terminus primarily by N332 (N-anchor) located in the helical domain. It moves approximately 4.0 Å (Cα) towards the catalytic domain upon peptide binding (Supplementary Fig. S2a). The distance between the N-anchor and the catalytic S652 permits accommodation of only two amino acid residues, revealing how the enzyme acquires its dipeptidyl peptidase specificity (Figs. 1c,d). Evolutionary conserved R670 is responsible for the Asp/Glu specificity at subsite S1: its guanidinium group directly interacts with the substrate carboxyl group of Asp/Glu (Fig. 1c, Supplementary Fig. S2b). R670 and R336 confer a dominant positive charge to subsite S1 further explaining its P1 acidic specificity (Supplementary Fig. S2c). Indeed, the substitution R670D completely abolished PeDPP11 activity^24^. In PeDPP11:LDVW, the third and fourth amino acids of the substrate (Val and Trp at positions P1′ and P2′, respectively) exhibit few interactions with the enzyme. For instance, Val (P1′) displays a weakly defined electron density with only 40% of its solvent accessible area buried by DPP11, while Trp (P2′) completely lacks electron density (Fig. 1d). This active site design renders the enzyme’s specificity more relaxed, with selectivity imposed mainly at P1 and P2 residues of the substrate. This promiscuous feature of DPP11 helps to provide nutrients for *P. gingivalis* and *P. endodontalis* given the scarce resources in the subgingival plaque^31^. However, the strategy to increase enzyme promiscuity comes with a price: the affinities for substrates and end products are strikingly similar (Fig. 2).

We performed a series of isothermal titration calorimetry experiments to further characterize peptide binding to PeDPP11. Binding of LD and RD dipeptides/end products to PeDPP11 was largely exothermic (Δ*H*_bind_ of −22.0 and −15.5 kJ.mol^−1^, respectively) at 25 °C indicating an enthalpy-driven process [Fig. 2 (left panel), Supplementary Fig. S3a]. A favourable change in entropy due to water displacement caused by peptide binding and concomitant domain motion was observed (Fig. 1b). Analysis of PeDPP11:RD using Naccess^32^ revealed a large loss of solvent-accessible area upon peptide binding, approximately 1430 Å^2^.

In contrast, binding of the LDVW and LDL substrates was largely endothermic (Δ*H*_bind_ of +23.8 and +17.0 kJ.mol^−1^, respectively) at 25 °C indicating an entropy-driven process to overcome the unfavourable enthalpic contribution [Fig. 2 (right panel), Supplementary Fig. S3b]. The binding of peptides to PgDPP11 induces the dimerization of its monomeric population (Supplementary Fig. S4), masking the real thermodynamic contributions involved in the binding process. In this way, we focused our thermodynamic analysis solely on PeDPP11.

Next, we asked what governs the opposite thermodynamic signatures observed for DPP11 binding of end products and substrates. To address this question, we dissected the contributions of the three possible components influencing the binding energetics: solvent, ligand and the protein itself. The most apparent answer would point to hydrophobic effects, which is the release of well-ordered water molecules from interfaces to the bulk solvent, resulting in system’s entropy increase upon ligand binding^33^. However, our crystal structures of DPP11 in complex with LDVW and dipeptide RD are both in closed conformation, excluding the possibility that solvent released from the protein’s cleft would explain the larger increase in entropy upon substrate binding. Then, we analysed the ligand’s contribution to the process. The presence of only one additional amino acid in the peptide LDL (Δ*H*_bind_ of +17.0 kJ.mol^−1^) compared to LD (Δ*H*_bind_ of −22.0 kJ.mol^−1^) results in the outstanding difference of +39.0 kJ.mol^−1^ in binding enthalpy. Due to the peptides similarity, energetic effects originating from the ligands alone do not suffice to explain the distinct thermodynamic binding forces reported. In light of the analyse above, we concluded that the major contribution for the opposite thermodynamic signatures must arise from the protein itself, via changes in conformational entropy, as demonstrated below.

**Figure 3:**
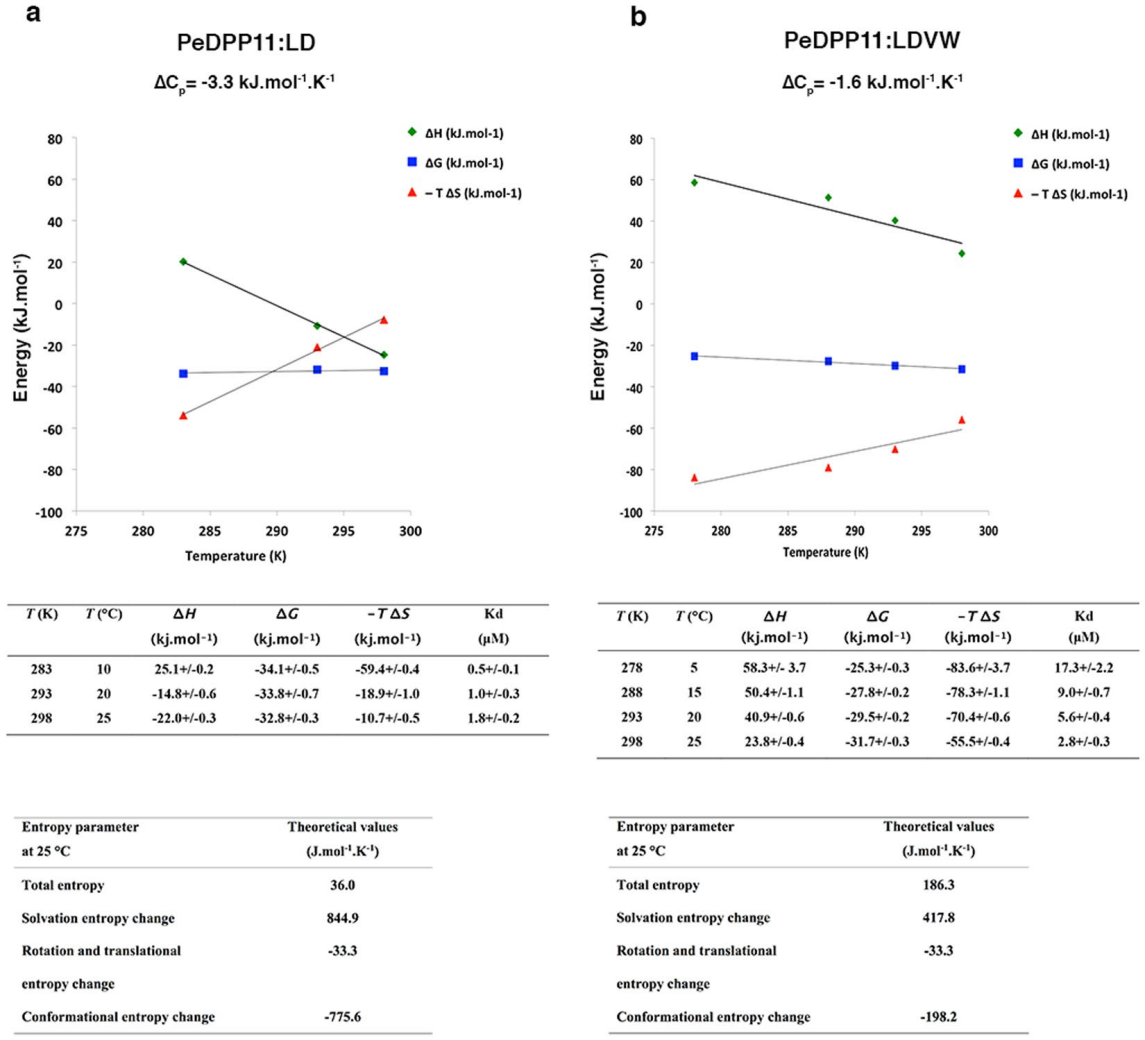
Thermodynamic analysis. **(a)** PeDPP11 binding to LD. **(b)** PeDPP11 binding to LDVW. Upper panels: Temperature dependence of ∆*G*, ∆*H* and −*T*∆S. Middle panel: Table with thermodynamic data derived from the ITC measurements at different temperatures. Lower panel: Entropy parameters estimations. Conformational entropy was calculated using the following equation: ∆*S*_conf_ = ∆*S*_tot_ − ∆*S*_sol_ − ∆*S*_rt_^34^. Where ∆*S*_sol_ = ∆*Cp* ln (298 K/385 K)^65^ and ∆*S*_rt_ is estimated using the “cratic entropy” value of −33.3 J.mol.^−1^K^−1^ ^66^.

In the free energy equation: Δ*G*_tot_ = Δ*H*_tot_ − *T*Δ*S*_tot_, the total binding entropy (Δ*S*_tot_) is deconvoluted into the sum of changes in Δ*S*_*conf*_ (conformational entropy), Δ*S*_sol_ (solvation entropy) and Δ*S*_RT_ (rotational and translational entropy)^34^. Based on experimentally-measured heat capacity changes (ΔC_p_) for PeDPP11:LDVW (−1.6 kJ.mol^−1^.K^−1^) and PeDPP11:LD (−3.3 kJ.mol^−1^.K^−1^) interactions, we calculated a Δ*S*_sol_ of +417.8 and +844.9 J.mol^−1^.K^−1^ and a Δ*S*_*conf*_ of −198.2 and −775.6 J.mol^−1^.K^−1^, respectively (Fig. 3). The data indicate that in both binding events the solvent provides a favourable contribution to the observed entropy and shows a 3.5-fold more prohibitive change in *overall* Δ*S*_*conf*_ for PeDPP11 interaction with LD compared to LDVW. We propose that the +677.3 J.mol^−1^.K^−1^ difference in Δ*S*_*conf*_ is associated with the unfolding of DPP11 specific regions upon binding to LDVW.

**Figure 4:**
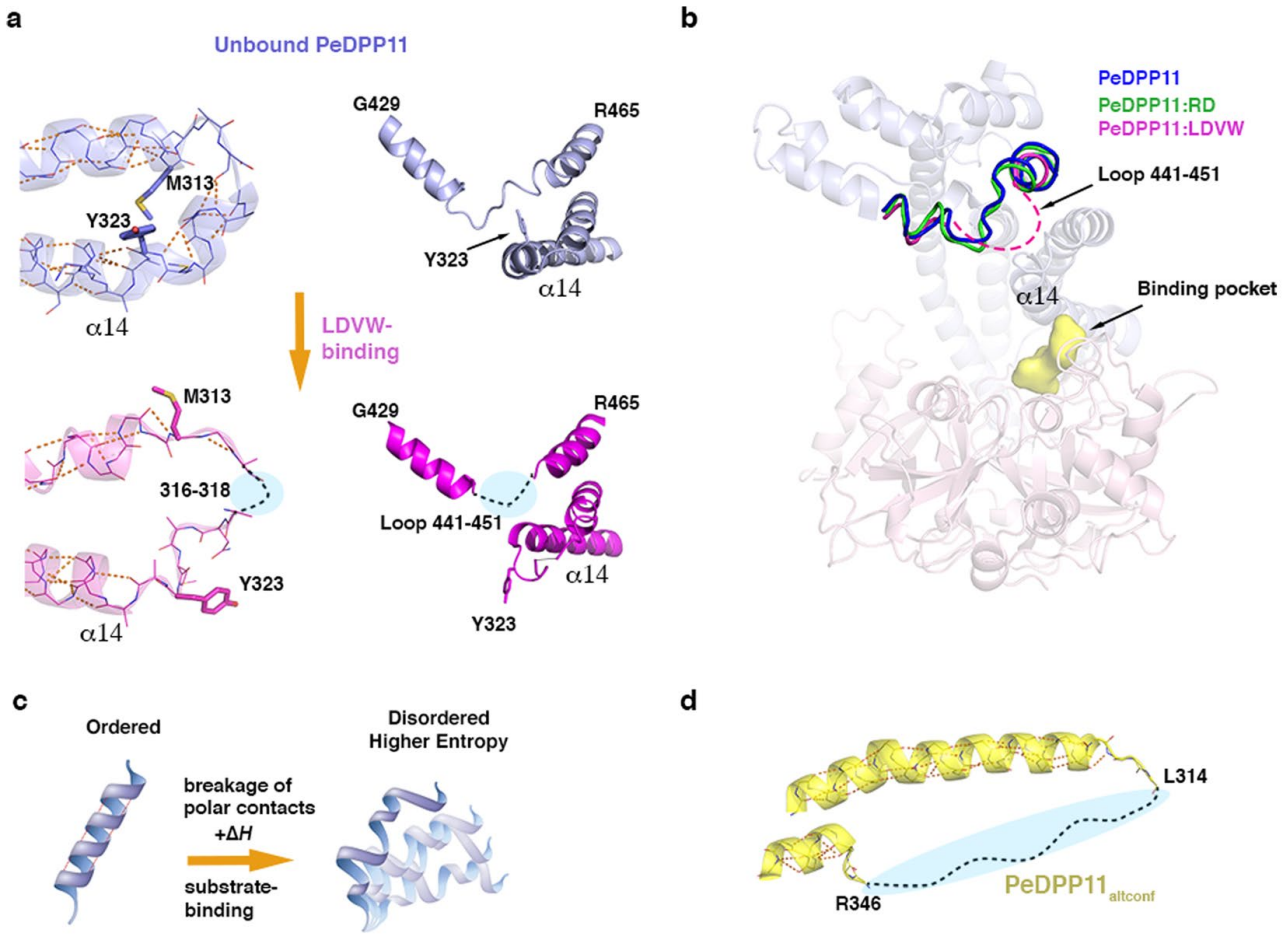
PeDPP11 conformational changes. **(a)** Close-up view of the main PeDPP11 regions that unfold upon binding to LDVW, as observed in the crystal structures. **(b)** Loop F441-K451 region superposition of unbound PeDPP11 (blue), PeDPP11:LDVW (magenta, dashed line) and PeDPP11:RD (green). Unbound PeDPP11 is represented as ribbons and peptide binding pocket as yellow surface. **(c)** Cartoon representation depicting a DPP11 helix unfolding. Upon substrate binding, energy is absorbed from the solution to break polar contacts, which causes helix destabilization. In the disordered stage, the helix accesses different structural states, increasing system entropy. **(d)** Close-up view of the helix α14 missing region in PeDPP11_altconf_. Intra-main chain polar contacts are indicated with orange dashed lines.

The helical domain displays a high diversity of structural states across all solved structures in this work. When compared to unbound PeDPP11, the r.m.s.d. of the helical domain is 5-fold higher than that of the catalytic domain for PeDPP11:LDVW and 2-fold higher for PeDPP11:RD and PeDPP11:RE (Supplementary Table S1a). Particularly, the unfolding of helix α14 (residues 320–346) and loop F441-K451 upon LDVW binding corroborates our hypothesis that protein conformational change is the determining factor for the opposed thermodynamics signatures observed upon peptide binding (Fig. 4a,b and Supplementary Fig. S5). We postulate that substrate binding leads to higher *protein* Δ*S*_*conf*_, which overcompensates for the unfavourable enthalpic contribution.

Consistent with the measured endothermic binding, we propose that energy is absorbed from the solution to break key interactions, such as those stabilizing loop F441-K451 and intra-main chain polar interactions that stabilize helix α14, but possibly in additional regions of the helical domain. These events permit the motion of the helical domain between different structural states leading to increased *protein* Δ*S*_*conf*_ (Fig. 4c). Usually, protein unfolding yields a positive ΔC_p_^35^, which explains the difference of +1.7 kJ.mol^−1^.K^−1^ in ΔC_p_ between PeDPP11:LDVW and PeDPP11:RD interactions. Upon DPP11-substrate binding, the increase in *protein* conformational entropy counterbalances the overall entropic costs in protein-peptide interactions (including loss of protein and peptide degrees of freedom). Interestingly, we obtained an unbound PeDPP11 crystal form, called here PeDPP11_altconf_, which lacks electron density for helix α14, indicating its susceptibility to unfold (Fig. 4d). Similar to PeDPP11 complexes, this structure is also closed (rotation angle of 27° of helical domain relative to the catalytic domain), illustrating the enzyme flexibility.

**Figure 5:**
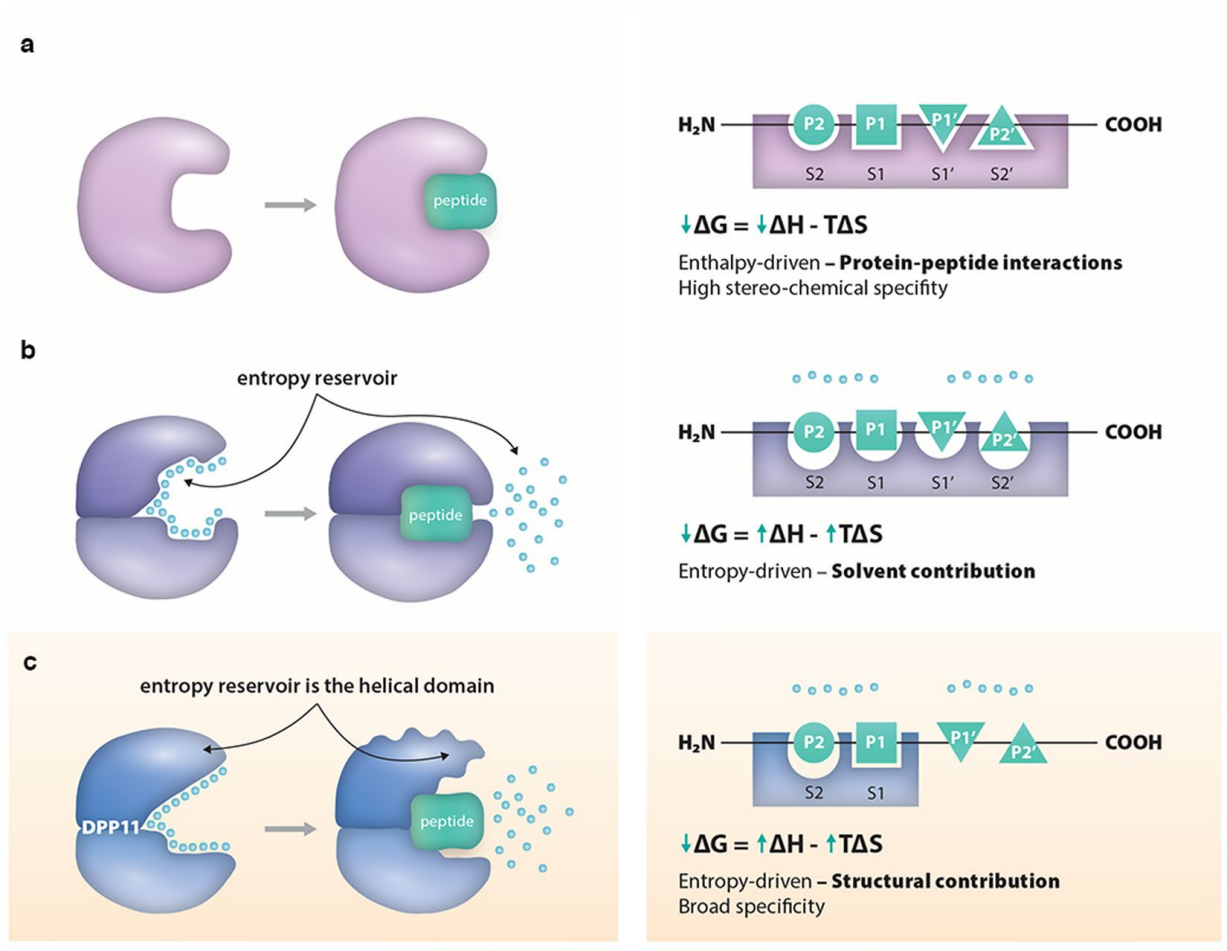
DPP11 conformational entropy in peptide binding. This cartoon illustrates two previously described models and DPP11 binding model reported in this work. **(a)** In the enthalpy-driven binding mode depicted, there are no major conformational changes and the active site is prearranged. The process is mainly governed by protein-peptide interactions, resulting in favourable enthalpy. **(b)** In this entropy-driven binding mode, the displacement of solvent molecules “entropy reservoir” provides the main driving-force for peptide-binding, and increases in system entropy outweighs the unfavourable enthalpy. In this case, peptide binding may be accompanied by protein conformational changes. **(c)** In DPP11 entropy-driven binding mode, protein conformational entropy is the main driving-force for substrate binding. De-structuring of parts of the helical domain “entropy reservoir” contributes to the increase in entropy necessary to compensate for the unfavourable enthalpy.

Protein-peptide interactions often occur in a way that minimizes the conformational changes of the protein partner, while maximizing their enthalpic potential via its packing and formation of hydrogen bonds^36^ (Fig. 5a). This strategy helps to decrease the entropic costs associated with the peptide loss of conformational entropy upon binding. The process can also be entropy-driven with the solvent providing the main driving-force, in this case, conformational flexibility may accompany peptide binding^37^ (Fig. 5b). Here, increased Δ*S*_*conf*_ in DPP11 establishes endothermic substrate binding via partial enzyme de-structuring associated with an increase in helical domain entropy, which acts as an “entropy reservoir” (Fig. 5c). DPP11 active site design displays stereochemical specificity only for P1 and P2 positions of the ligand. This arrangement favours substrate entropy-driven binding by limiting the enthalpic contributions of protein-peptide interaction (i.e. limiting the number of polar contacts) for only the two first amino acids of the incoming peptide. Additionally, our data also illustrate how conformational plasticity enables enzyme promiscuity; for instance, by closing differently around different ligands^38^.

Due to experimental challenges, the role of conformational entropy in molecular recognition by proteins has begun to be elucidated only recently, mainly by nuclear magnetic resonance (NMR) relaxation methods^39^. Using NMR techniques and molecular dynamics simulations, Veglia and colleagues observed in cAMP-dependent protein kinase A (PKA-C) a similar binding mode to that of DPP11. They showed that the substrate PLN_1-20_ (phospholamban) binds to (PKA-C) in an entropically driven way, resulting in protein increased conformational dynamics. Conversely, binding of the inhibitor PKI_5-24_ (protein kinase inhibitor) to PKA-C is enthalpically driven and stabilizes the protein, quenching the enzyme dynamics, which is important to prime the active for catalysis^40^.

The structural and thermodynamics data presented here provide a distinct model for protein-protein interaction, particularly in cases where increase in protein conformational entropy significantly contributes to the free energy of binding. Together with PKA-C, DPP11 binding mode may represent a general mechanism for biomolecular recognition, allowing the identification of proteins that share similar features and that have evolved to promiscuously bind numerous ligands. These findings further provide an innovative framework for structure-based drug design to develop compounds that target the “entropy reservoirs”. For instance, molecules able to prevent the unfolding of helix 14 and loop F441-K451 could display efficient inhibitor properties. Alternatively, it is also conceivable the identification of effectors that increase catalytic power by promoting protein dynamics.

## Methods

### Protein expression and purification

*E. coli* codon-optimized genes encoding for C-terminal 6xHis-tagged PeDPP11_22-717_ S652A and PgDPP11_22-720_ S655A in the pET-22b(+) vector (cloning sites *N*deI and *X*hoI) were purchased from GenScript (Piscataway, USA). The construct *Flavobacterium psychrophilum* DPP11 (called here FpDPP11_17-713_) encoding the N-terminal fusion sequence (MGGSHHHHHHGMASMTGGQQMGRDLYDDDDKDPTL) was cloned into the expression vector pTricHis.

All plasmids were transformed in BL21(DE3)pLysS. The cells were grown in LB-medium containing 100 μg ml^−1^ ampicillin and 34 μg ml^−1^ chloramphenicol. After 3 h at 37 °C, temperature was reduced to 30 °C and protein expression was induced by the addition of 0.5 mM isopropyl-1-thio-D-galactopyranoside (IPTG). Cells were then allowed to grow for 4 h and were harvested by centrifugation at 4,000 *g* for 10 minutes. For protein purification, cells were resuspended in 50 mM Hepes-NaOH pH 8.0, 150 mM NaCl. Cell debris was removed by centrifugation at 25,000 *g* for 45 minutes at 4 °C, and the supernatant was subjected to affinity chromatography on 5 ml HisTrap^TM^ (GE Healthcare) equilibrated with lysis buffer. Bound protein was eluted in lysis buffer containing 500 mM imidazole. Further purification was performed by size exclusion chromatography (SEC) on a HiLoad 26/60 Superdex 200 (GE Healthcare) column previously equilibrated with 10 mM Hepes-NaOH pH 7.4, 100 mM NaCl. Purified protein was concentrated using 20 ml concentrators with an appropriate molecular weight cut-off (Vivaspin® 50,000 MWCO, Sartorius).

### Crystallization

To enhance the crystallizability of PgDPP11 and PeDPP11, truncated forms of the enzymes were designed lacking the first 21 amino acid residues (called here PgDPP11_22-720_ and PeDPP11_22-717_) which were predicted to be signal peptides^41^. The following crystallization trials used the nanodrop-dispensing robot (Phoenix RE; Rigaku Europe) employing the sitting drop vapour diffusion technique by mixing equal volumes of protein (200 nl) and reservoir solutions (200 nl) at 20 °C in a 96-well Intelli-Plate (ArtRobbins Instruments®). All crystals were cryoprotected in a solution consisting of reservoir solution supplemented with 20% glycerol before flash-cooling in liquid nitrogen. X-ray diffraction data were collected at 100 K.

- **PgDPP11**_**22-720**_ **S655A (PgDPP11)** was crystallized at 10 mg ml^−1^ using the Morpheus^42^ screen condition D11: 0.12 M alcohols, buffer system 3 pH 8.5, 40% v/v glycerol, 20% w/v PEG 4000.
- **PeDPP11**_**22-717**_ **S652A (PeDPP11:RD)** at 10 mg ml^−1^ was incubated with 1.2 mM dipeptide Arg-Asp on ice for 15 minutes. Crystals were obtained in the Morpheus screen condition E10: 0.12 M ethylene glycols, 0.1 M buffer system 3 pH 8.5, 40% v/v ethylene glycol, 20% w/v PEG 8000.
- **PeDPP11**_**22-717**_ **S652A (PeDPP11:RE)** at 10 mg ml^−1^ was incubated with 1.2 mM dipeptide Arg-Glu on ice for 15 minutes. Crystals were obtained in the Morpheus screen condition F12: 0.12 M monosacharides, 0.1 M buffer system 3 pH 8.5, 25% v/v MPD, 25% PEG 1000, 25% w/v PEG 3350.
- **FpDPP11**_**17-713**_ was crystallized at 10 mg ml^−1^ in the PACT Premier screen (Molecular Dimensions®) condition G2: 0.2 M NaBr, 0.1 M bistris propane, pH 7.5, 20% PEG 3350.

The following crystallization trials used the sitting-drop vapour diffusion method and were conducted at the High Throughput Crystallization Laboratory of the EMBL Grenoble Outstation (https://embl.fr/htxlab)^43^. Drops of 100 nl sample and 100 nl crystallization solution were set up in CrystalDirect plates (MiTeGen, Ithaca, USA) with a Cartesian PixSys robot (Cartesian Technologies, Irvine, USA). The experiments were incubated at 20 °C in a RockImager system (Formulatrix Inc., Bedford, USA). Automated high-throughput crystal cryo-cooling and harvesting were performed with CrystalDirect^TM^ Technology as described by Zander *et al*., 2016^44^. Crystals were stored in liquid nitrogen for data collection. Data collection was done in a fully automated fashion at MASSIF-1, ESRF^45^. X-ray diffraction data were collected at 100 K.

- **PeDPP11**_**22-717**_ **S652A (unbound)** was crystallized at 10 mg ml^−1^ initially in the condition D11 of ProComplex screen (Qiagen®). The condition was further optimized to 0.1 M Tris-HCl pH 7.5, 15% PEG 6000.
- **PeDPP11**_**22-717**_ **S652A in the alternate conformation (PeDPP11**_**altconf**_ **)** was crystallized initially in the condition B2 of ProComplex screen (Qiagen®): 0.1 M calcium acetate, 10% w/v PEG 4000, 0.1 M sodium acetate pH 4.5. The condition was further optimized to: 0.1 M calcium acetate, 15% w/v PEG 4000, 0.1 M sodium acetate pH 5.0.
- **PeDPP11**_**22-717**_ **S652A (PeDPP11:LDVW)** at 22 mg ml^−1^ was incubated for 30 minutes on ice with 1.0 mM Leu-Asp-Val-Trp. Crystals were initially obtained in condition D11 of ProComplex screen (Qiagen®). The condition was further optimized to 0.1 M Tris-HCl pH 7.5, 15% PEG 6000.

### Structure determination

Our initial attempts to solve the structure by molecular replacement using the coordinates of dipeptidyl aminopeptidase BII (DAP BII)^46^ from *Pseudoxanthomonas mexicana* WO24, the closest homologue (37% identity) with known 3D structure, were ineffective. We suspected that different conformations adopted by the protein in the crystal could be rendering molecular replacement trials unsuccessful. So, a DPP11 homologue (37% identical to PgDPP11) from FpDPP11_17-713_ was employed to grow monoclinic crystals (space group *P*2_1_). FpDPP11_17-713_ structure was then determined by molecular replacement using the coordinates of DAP BII (PDB code: 3WOJ, 27% identity). Subsequently, FpDPP11_17-713_ structure was successfully used as template to solve the structures of PgDPP11_22-720_ S655A and PeDPP11_22-717_ S652A (Supplementary Fig. S6). Two out of four subunits in the crystal asymmetric unit of FpDPP11_17-713_ were in complex with Arg-Asp, a dipeptide co-purified from *E. coli*. This finding led us to grow co-crystals of PeDPP11_22-717_ S652A in complex with dipeptides Arg-Asp and Arg-Glu.

Data were processed with XDS^47^, Scala^48^ and Pointless^49^. All structures were solved by molecular replacement with PHASER^50^, refined with PHENIX^51^ and manually adjusted in COOT^52^. R_free_-values^53^ were computed from 5% randomly chosen reflections not used for the refinement. The structure stereochemistry was checked using Molprobity^54^. Details of data collection and refinement statistics are provided in Table 1. Peptide omit maps are depicted in Supplementary Fig. S7. All figures were prepared using the program PyMOL (http://www.pymol.org). Poisson-Boltzmann calculations were performed using the software APBS^55, 56^.

### Isothermal Titration Calorimetry

All experiments were carried out in 10 mM Hepes-NaOH pH 7.4, 100 mM NaCl. Both the enzymes and the peptides were dissolved in the same buffer. The bindings were analysed with a MicroCal^TM^ iTC_200_ microcalorimeter (GE Healthcare, Life Sciences) equilibrated at the respective temperature. Typically, a total of one aliquot of 0.4 μl and 19 aliquots of 2.0 μl of the peptide solution were injected at a rate of 0.5 μl/s into 200 μl of the protein solution under constant stirring at 750 rpm at the specified concentrations. The following titrations were performed: 600 μM LDVW to 60 μM PeDPP11, 810 μM LD to 75 μM PeDPP11 (at 10 °C, 1.28 mM LD to 120 μM PeDPP11 was employed), 1 mM of LDL to 75 μM PeDPP11, 1.0 mM of RD to 80 μM M PeDPP11. As a control to exclude buffer-dependent effects, we additionally performed the binding of 600 μM LDVW to 60 μM PeDPP11 and 600 μM LD to 75 μM PeDPP11 in 50 mM sodium phosphate pH 7.4 with 100 mM NaCl (Supplementary Fig. S8). Every injection was carried out over a period of 4 s with a spacing of 110 s between the injections. The corresponding heats of binding were determined by integrating the observed peaks after correcting for the heat dilution of the peptide determined in a reference measurement (peptide injected into buffer). These corrected values were plotted against the ratio peptide *vs*. protein concentration in the cell to generate the binding isotherm. Nonlinear least-squares fitting using Origin version 7.0 (Microcal) was used to obtain the association constants (*K*_a_), heats of binding (Δ*H*) and stoichiometries. *K*_d_ and Gibbs free energy (Δ*G*) were calculated according to: *K*_d_ = 1/*K*_a_ and Δ*G* = −R*T* ln *K*_a_ = R*T* ln *K*_d_. The reported values are averages of at least two independent measurements. The stoichiometry obtained in all experiments is within the range 0.7–1.1, which is in agreement with the crystal structures (stoichiometry 1).

### Small-angle X-ray scattering (SAXS) data collection and analysis

SAXS experiments were performed at 0.9918 Å wavelength ESRF at BioSAXS beamline BM29 (Grenoble, France) equipped with PILATUS 1 M^57^. The detector distance was set at 2.864 m. The range of scattering vector 0.03 nm^−1^ < q < 4.5 nm^−1^ was covered. For PeDPP11_22-717_ S652A, the data were collected using the following protein concentrations: 1.1 mg ml^−1^, 1.9 mg ml^−1^, 9.62 mg ml^−1^ and 16.37 mg ml^−1^.

The samples were in a buffer containing 10 mM Hepes-NaOH pH 7.4, 100 mM NaCl, and the measurements were performed at 20 °C. The automated sample changer^58^ was employed to load the samples and constantly remove the irradiated sample. Twenty successive exposures of 1 second were collected and compared to detect and discard possible radiation damage effects. For PgDPP11_22-720_ S655A, on-line HPLC-mode was used with a Superdex^TM^ 200 10/300 GL column (GE Healthcare), and SAXS data was recorded directly on the sample eluted. A 0.5 ml min^−1^ flow was used. The protein concentration applied onto the column was 50 mg ml^−1^.

The data were processed and analyzed using the online analysis pipeline^59^. Subsequent manual processing was done with the ATSAS 2.6 program package^60^. The forward scattering I(0) and the radius of gyration Rg were extracted from the Guinier approximation calculated with the AutoRG function within PRIMUS^61, 62^. The maximum particle dimension *D*max and P(r) function were evaluated using the program GNOM^63^. For PeDPP11_22-717_ S652A, the analysis of SAXS data by Guinier approximation showed no concentration dependence effect, indicating the samples were homogeneous and free of aggregation. For this construct, SAXS analyses were performed by merging data from all concentrations measured. For both PeDPP11_22-717_ S652A and PgDPP11_22-720_ S655A, the theoretical scattering from the crystallographic structures was calculated using the program CRYSOL^64^ and compared with the respective scattering profiles.

### Size exclusion chromatography followed by Multiangle Laser Light Scattering

To assess the oligomeric state and molecular weight, the samples were applied onto a Superdex^TM^ 200 10/300 GL column (GE Healthcare) at the respective concentrations, using a flow of 0.5 ml/min. The column was connected to a miniDAWN Tristar light scattering instrument (Wyatt Technologies, Santa Barbara, CA) and pre-equilibrated with 10 mM Hepes-NaOH pH 7.4, 100 mM NaCl. Data analysis was performed using the manufacturer’s software ASTRA.

### Hydrolyzing activity toward MCA-dipeptides

Purification of recombinant active forms of PgDPP11 and PeDPP11 was performed according to Ohara-Nemoto *et al*.^24^. PgDPP11 and PeDPP11 (2-20 ng) were used for measurement of dipeptidyl peptidase activity in 200 μl of reaction solution composed of 50 mM sodium phosphate pH 7.0 and 5 mM EDTA. The reaction was started with an addition of 20 μM Leu-Asp-, Arg-Asp or Leu-Glu-MCA and continued at 37 °C for 30 min (Supplementary Fig. S9). Fluorescence intensity was measured with excitation at 380 nm and emission at 460 nm with a Fluorescence Photometer F-4000 (Hitachi).

## Electronic supplementary material

Supplementary Information

## References

[1] Eke PI (2015). Update on Prevalence of Periodontitis in Adults in the United States: NHANES 2009 to 2012. J. Periodontol..

[2] Batchelor P (2014). Is periodontal disease a public health problem?. Br. Dent. J..

[3] Chee B, Park B, Bartold PM (2013). Periodontitis and type II diabetes: a two-way relationship. Int. J. Evid. Based Healthc..

[4] Hajishengallis G, Darveau RP, Curtis MA (2012). The keystone-pathogen hypothesis. Nat. Rev. Microbiol..

[5] Lombardo Bedran TB (2012). Porphyromonas endodontalis in chronic periodontitis: a clinical and microbiological cross-sectional study. J. Oral Microbiol..

[6] Wang J, Jia H (2016). Metagenome-wide association studies: fine-mining the microbiome. Nat. Rev. Microbiol..

[7] Lalla E, Papapanou PN (2011). Diabetes mellitus and periodontitis: a tale of two common interrelated diseases. Nat. Rev. Endocrinol..

[8] Lundberg K, Wegner N, Yucel-Lindberg T, Venables PJ (2010). Periodontitis in RA-the citrullinated enolase connection. Nat. Rev. Rheumatol..

[9] Javed F, Warnakulasuriya S (2015). Is there a relationship between periodontal disease and oral cancer? A systematic review of currently available evidence. Crit. Rev. Oncol. Hematol..

[10] Ha NH (2015). Prolonged and repetitive exposure to Porphyromonas gingivalis increases aggressiveness of oral cancer cells by promoting acquisition of cancer stem cell properties. Tumour Biol..

[11] Ruiz IF (2016). Risk factors: Periodontitis increases risk of a first MI. Nat. Rev. Cardiol..

[12] Farhad SZ (2014). The effect of chronic periodontitis on serum levels of tumor necrosis factor-alpha in Alzheimer disease. Dent. Res. J. (Isfahan).

[13] Paju S, Scannapieco FA (2007). Oral biofilms, periodontitis, and pulmonary infections. Oral Dis..

[14] Hajishengallis G (2015). Periodontitis: from microbial immune subversion to systemic inflammation. Nat. Rev. Immunol..

[15] Rouf SM (2013). Phenylalanine 664 of dipeptidyl peptidase (DPP) 7 and Phenylalanine 671 of DPP11 mediate preference for P2-position hydrophobic residues of a substrate. FEBS Open Bio.

[16] Nemoto TK, Ohara-Nemoto Y (2016). Exopeptidases and gingipains in Porphyromonas gingivalis as prerequisites for its amino acid metabolism. Jpn. Dent. Sci. Rev..

[17] Takahashi N (2015). Oral Microbiome Metabolism: From “Who Are They?” to “What Are They Doing?”. J. Dent. Res..

[18] Xu Q (2016). A Distinct Type of Pilus from the Human Microbiome. Cell.

[19] Holt SC, Kesavalu L, Walker S, Genco CA (1999). Virulence factors of Porphyromonas gingivalis. Periodontol. 2000.

[20] de Diego I (2014). Structure and mechanism of cysteine peptidase gingipain K (Kgp), a major virulence factor of Porphyromonas gingivalis in periodontitis. J. Biol. Chem..

[21] Nelson KE (2003). Complete genome sequence of the oral pathogenic Bacterium porphyromonas gingivalis strain W83. J. Bacteriol..

[22] Ohara-Nemoto Y (2014). Identification and characterization of prokaryotic dipeptidyl-peptidase 5 from Porphyromonas gingivalis. J. Biol. Chem..

[23] Takahashi N, Sato T, Yamada T (2000). Metabolic pathways for cytotoxic end product formation from glutamate- and aspartate-containing peptides by Porphyromonas gingivalis. J. Bacteriol..

[24] Ohara-Nemoto Y (2011). Asp- and Glu-specific novel dipeptidyl peptidase 11 of Porphyromonas gingivalis ensures utilization of proteinaceous energy sources. J. Biol. Chem..

[25] Kurita-Ochiai T (2008). Butyric acid induces apoptosis in inflamed fibroblasts. J. Dent. Res..

[26] Rouf SM (2013). Discrimination based on Gly and Arg/Ser at position 673 between dipeptidyl-peptidase (DPP) 7 and DPP11, widely distributed DPPs in pathogenic and environmental gram-negative bacteria. Biochimie.

[27] Sakamoto Y (2015). Structural and mutational analyses of dipeptidyl peptidase 11 from Porphyromonas gingivalis reveal the molecular basis for strict substrate specificity. Sci. Rep..

[28] Polgar L (2005). The catalytic triad of serine peptidases. Cell. Mol. Life Sci..

[29] Hayward S, Berendsen HJ (1998). Systematic analysis of domain motions in proteins from conformational change: new results on citrate synthase and T4 lysozyme. Proteins.

[30] Hayward S, Lee RA (2002). Improvements in the analysis of domain motions in proteins from conformational change: DynDom version 1.50. J. Mol. Graph. Model..

[31] Kuboniwa M, Lamont RJ (2010). Subgingival biofilm formation. Periodontol. 2000.

[32] Naccess 2.1.1 v. 2.1.1 (Department of Biochemistry and Molecular Biology, University College, London., 1996).

[33] Biela A (2012). Ligand bindin g stepwise disrupts water network in thrombin: enthalpic and entropic changes reveal classical hydrophobic effect. J. Med. Chem..

[34] Marlow MS, Dogan J, Frederick KK, Valentine KG, Wand AJ (2010). The role of conformational entropy in molecular recognition by calmodulin. Nat. Chem. Biol..

[35] Prabhu NV, Sharp KA (2005). Heat capacity in proteins. Annu. Rev. Phys. Chem..

[36] London N, Movshovitz-Attias D, Schueler-Furman O (2010). The structural basis of peptide-protein binding strategies. Structure.

[37] Bezerra GA (2012). Entropy-driven binding of opioid peptides induces a large domain motion in human dipeptidyl peptidase III. Proc. Natl. Acad. Sci. USA.

[38] Nobeli I, Favia AD, Thornton JM (2009). Protein promiscuity and its implications for biotechnology. Nat. Biotechnol..

[39] Frederick KK, Marlow MS, Valentine KG, Wand AJ (2007). Conformational entropy in molecular recognition by proteins. Nature.

[40] Masterson LR (2011). Dynamically committed, uncommitted, and quenched states encoded in protein kinase A revealed by NMR spectroscopy. Proc. Natl. Acad. Sci. USA.

[41] Slabinski L (2007). XtalPred: a web server for prediction of protein crystallizability. Bioinformatics.

[42] Gorrec F (2009). The MORPHEUS protein crystallization screen. J. App. Cryst..

[43] Dimasi N, Flot D, Dupeux F, Marquez JA (2007). Expression, crystallization and X-ray data collection from microcrystals of the extracellular domain of the human inhibitory receptor expressed on myeloid cells IREM-1. Acta Crystallogr. Sect. F Struct. Biol. Cryst. Commun..

[44] Zander U (2016). Automated harvesting and processing of protein crystals through laser photoablation. Acta Cryst. D.

[45] Svensson O, Malbet-Monaco S, Popov A, Nurizzo D, Bowler MW (2015). Fully automatic characterization and data collection from crystals of biological macromolecules. Acta Cryst. D.

[46] Sakamoto Y (2014). S46 peptidases are the first exopeptidases to be members of clan PA. Sci. Rep..

[47] Kabsch WX (2010). Acta Cryst. D.

[48] Evans P (2006). Scaling and assessment of data quality. Acta Cryst. D.

[49] Evans PR (2011). An introduction to data reduction: space-group determination, scaling and intensity statistics. Acta Cryst. D.

[50] McCoy AJ (2007). Phaser crystallographic software. J. App. Cryst..

[51] Adams PD (2011). The Phenix software for automated determination of macromolecular structures. Methods.

[52] Emsley P, Cowtan K (2004). Coot: model-building tools for molecular graphics. Acta Cryst. D.

[53] Kleywegt GJ, Brunger AT (1996). Checking your imagination: applications of the free R value. Structure.

[54] Chen VB (2010). MolProbity: all-atom structure validation for macromolecular crystallography. Acta Cryst. D.

[55] Baker NA, Sept D, Joseph S, Holst MJ, McCammon JA (2001). Electrostatics of nanosystems: application to microtubules and the ribosome. Proc. Natl. Acad. Sci. USA.

[56] Dolinsky TJ, Nielsen JE, McCammon JA, Baker NA (2004). PDB2PQR: an automated pipeline for the setup of Poisson-Boltzmann electrostatics calculations. Nucleic Acids Res..

[57] Pernot P (2013). Upgraded ESRF BM29 beamline for SAXS on macromolecules in solution. J. Synchrotron Rad..

[58] Round A (2015). BioSAXS Sample Changer: a robotic sample changer for rapid and reliable high-throughput X-ray solution scattering experiments. Acta Cryst. D.

[59] Brennich ME (2016). Online data analysis at the ESRF bioSAXS beamline, BM29. J. App. Cryst..

[60] Petoukhov MV (2012). New developments in the program package for small-angle scattering data analysis. J. App. Cryst..

[61] Petoukhov MV, Konarev PV, Kikhney AG, Svergun DI (2007). ATSAS 2.1 - towards automated and web-supported small-angle scattering data analysis. J. App. Cryst..

[62] Konarev PV, Volkov VV, Sokolova AV, Koch MHJ, Svergun DI (2003). PRIMUS: a Windows PC-based system for small-angle scattering data analysis. J. App. Cryst..

[63] Svergun D (1992). Determination of the regularization parameter in indirect-transform methods using perceptual criteria. J. App. Cryst..

[64] Svergun D, Barberato C, Koch MHJ (1995). CRYSOL - a Program to Evaluate X-ray Solution Scattering of Biological Macromolecules from Atomic Coordinates. J. App. Cryst..

[65] Baldwin RL (1986). Temperature dependence of the hydrophobic interaction in protein folding. Proc. Natl. Acad. Sci. USA.

[66] Murphy KP, Xie D, Thompson KS, Amzel LM, Freire E (1994). Entropy in biological binding processes: estimation of translational entropy loss. Proteins.

