# Supplementary material for: Bacterial protease uses distinct thermodynamic signatures for substrate recognition
Source: Sci Rep. 2017 Jun 6;7:2848. doi: 10.1038/s41598-017-03220-y (PMC5460201; doi:10.1038/s41598-017-03220-y)
Supplement: Supplementary file 1 — Supplementary Information [file 41598_2017_3220_MOESM1_ESM.pdf]

Bacterial protease uses distinct thermodynamic signatures for substrate recognition

Gustavo Arruda Bezerra, Yuko Ohara-Nemoto, Irina Cornaciu, Sofiya Fedosyuk, Guillaume Hoffmann, Adam Round, José A. Márquez, Takayuki K. Nemoto, Kristina Djinovic-Carugo

Contents:

Supplementary Figures 1-9

Supplementary Tables 1-2

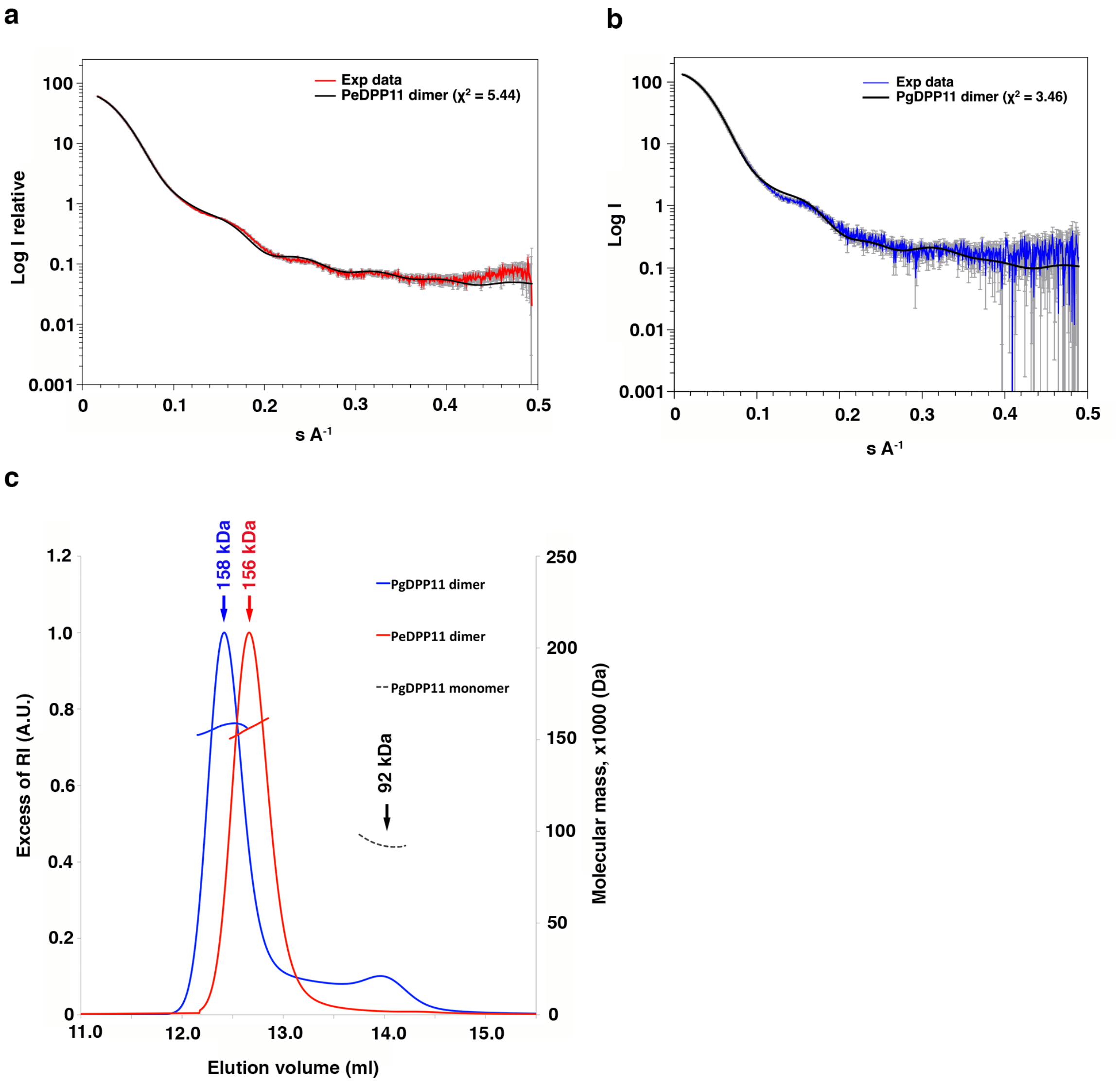

Supplementary Figure S1. DPP11 oligomeric state analysis. (a) The SAXS theoretical scattering profile (black line) of PeDPP11 homodimer is compared to the experimental scattering profile (red lines). **(b)** The SAXS theoretical scattering profile (black line) of PgDPP11 homodimer is compared to the experimental scattering profile (blue lines). **(c)** SEC-MALLS profile of PeDPP11 (red line) and PgDPP11 (blue line). All samples were applied at 3 mg/ml onto a Superdex 10/300 GL column pre-equilibrated with 10 mM Hepes-NaOH pH 7.4, 100 mM NaCl. Protomers theoretical molecular weight: PeDPP11= 79645.3 Da and PgDPP11 = 80556.3 Da.

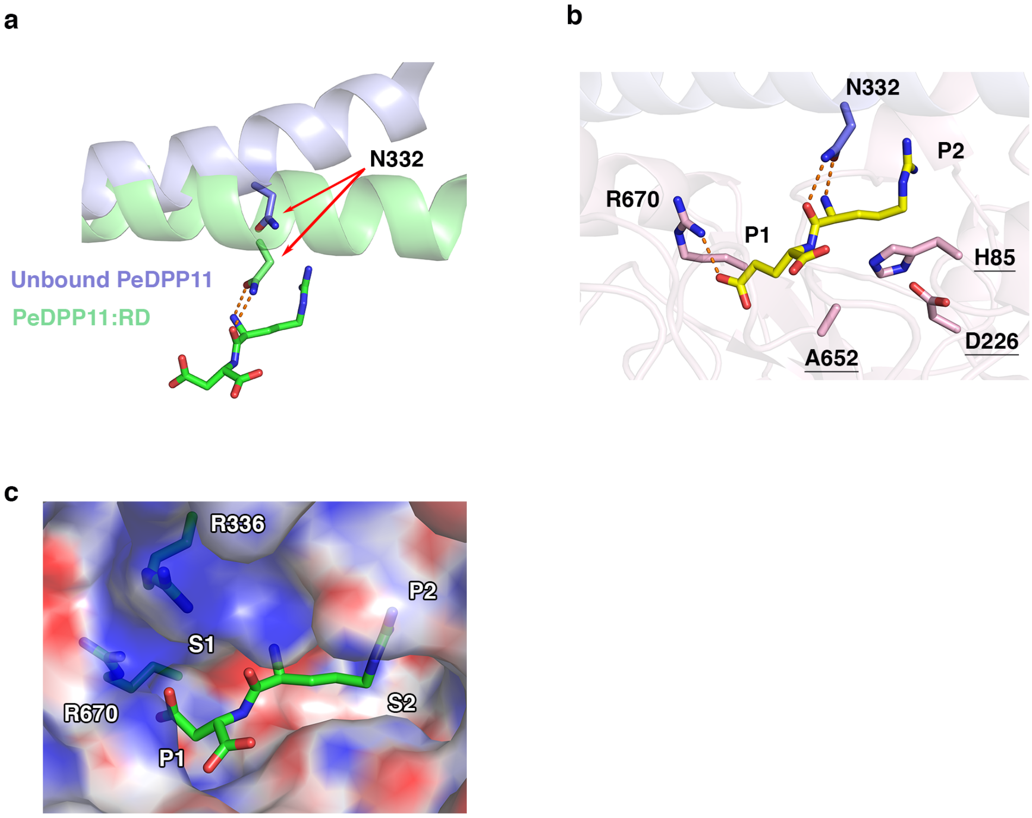

Supplementary Figure S2. Active site of *Porphyromonas endodontalis* DPP11. (a) Asn332 (N-anchor) movement (its Cα moves about 4.0 Å) upon peptide binding. The peptide RD is represented as green sticks. **(b)** Active site of PeDPP11:RE (shown in yellow). Catalytic triad is underlined. Note that S652 is mutated to alanine. **(c)** PeDPP11 electrostatic potential. Surface representation of PeDPP11 Poisson-Boltzmann electrostatic potential of the peptide binding pocket. Peptide Arg-Asp is shown as green sticks. R670 and R336 (shown as green sticks) are the main residues contributing to S1 subsite charge distribution.

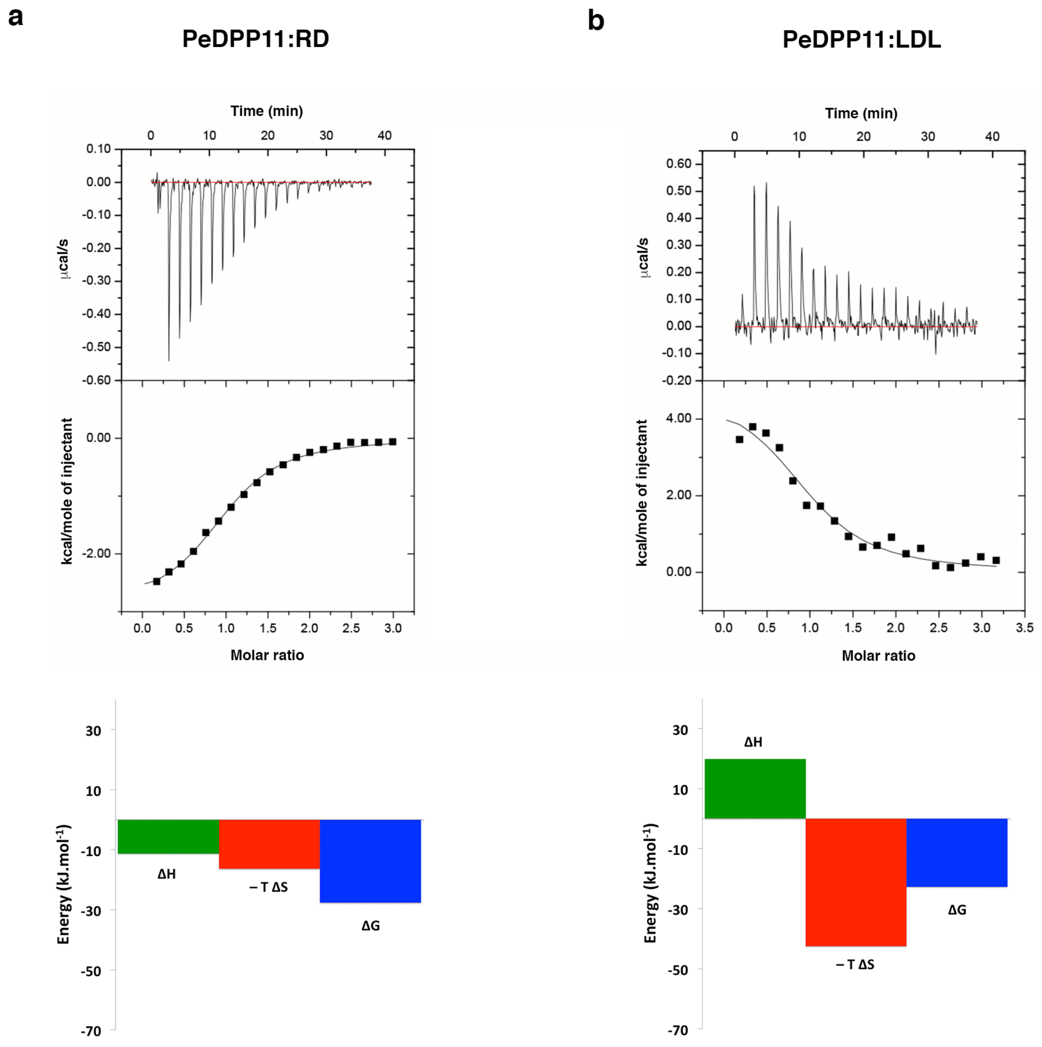

Supplementary Figure S3. Microcalorimetric analysis. Isothermal titration calorimetry experiments performed by titrating RD **(left panel)** and LDL **(right panel)** into PeDPP11. Upper panel shows time-dependent deflection of heat for each injection (top). Integrated calorimetric data for the respective interactions (bottom). The continuous curve represents the best fit using a one-site binding model. Lower panel shows the graphical representation of thermodynamics parameters.

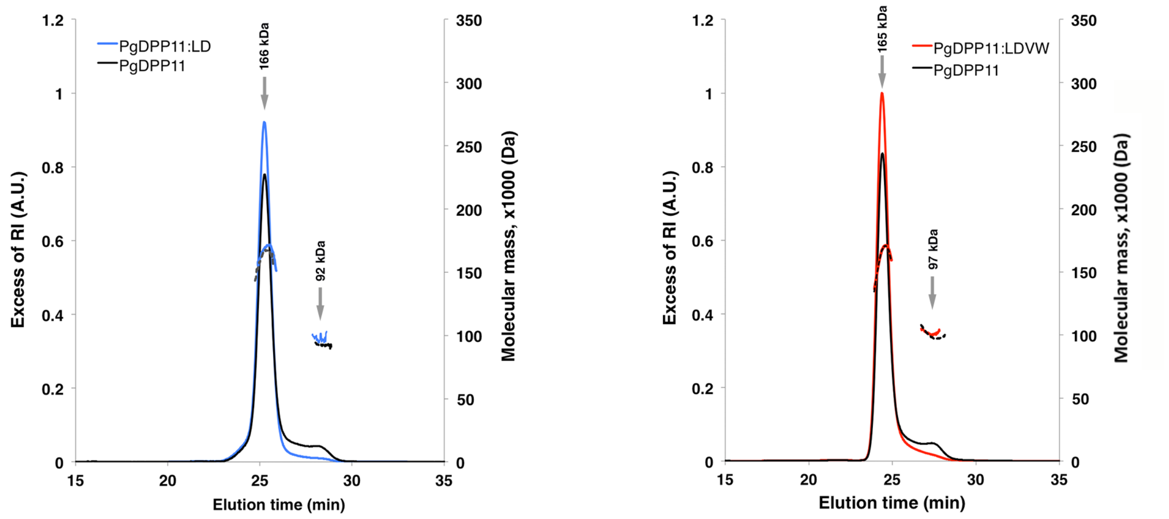

Supplementary Figure S4. Analysis of *Porphyromonas gingivalis* DPP11 binding to LD and LDVW. PgDPP11 partial peptide-induced dimerization. **Left side:** SEC-MALLS profile of unbound 83 μM PgDPP11 (black line) and 83 μM PgDPP11: 280 μM LD (green line). **Right side:** SEC-MALLS profile of unbound 83 μM PgDPP11 (black line) and 83 μM PgDPP11: 1400 μM LDVW (red line). All samples were applied onto a Superdex 10/300 GL column pre-equilibrated with 10 mM Hepes-NaOH pH 7.4, 100 mM NaCl. Upon peptide binding, PgDPP11 monomeric fraction is shifted to the dimeric fraction.

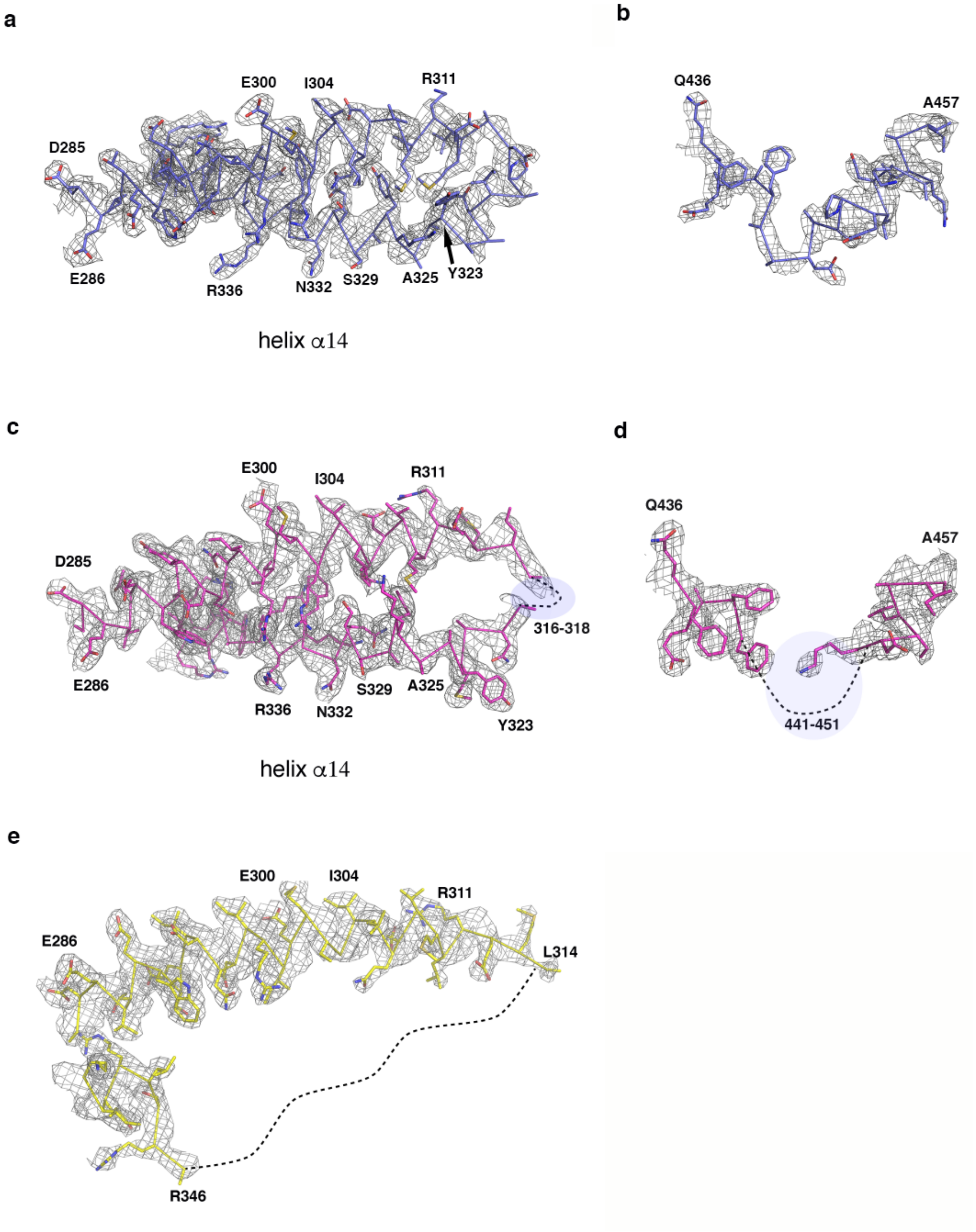

Supplementary Figure S5. 2Fo-Fc electron density map (σ=1) of PeDPP11 "entropy reservoir" regions. (a) Close-up view of unbound PeDPP11 helix α14 and **(b)** loop 441-451 region. **(c)** Close-up view of PeDPP11:LDVW helix α14 and **(d)** loop 441-451 region. **(e)** Close-up view of PeDPP11$_{altconf}$ helix α14 region.

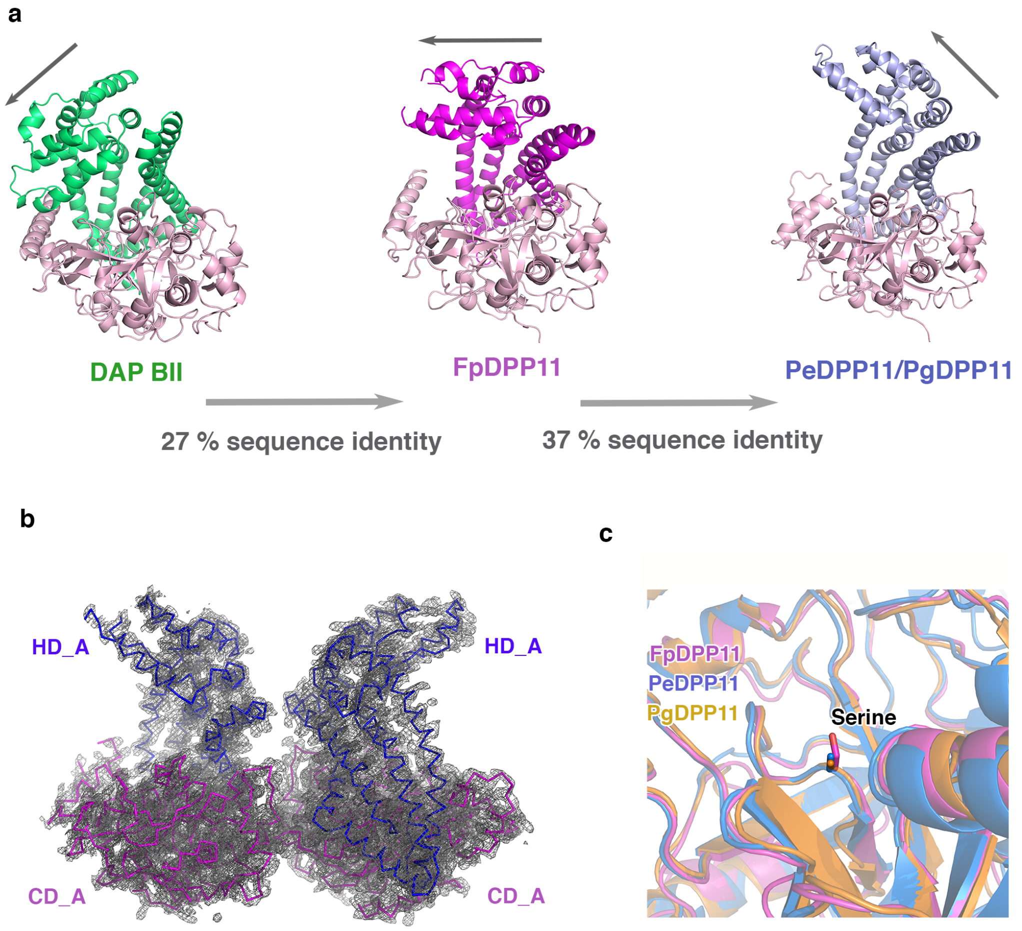

Supplementary Figure S6. Comparison of DAP BII, FpDPP11, PeDPP11 and PgDPP11. (a) Orientation of the helical domain relative to the catalytic domain of DAP BII (green), FpDPP11 (magenta) and PeDPP11/PgDPP11 (blue). Catalytic domain is shown in pink. The arrows indicate the helical domain orientation relative to the catalytic domain. FpDPP11 helical domain adopts an intermediate conformation compared to DAP BII and PeDPP11/PgDPP11. **(b)** 2Fo-Fc electron density map (σ=1) in one asymmetric unit of PeDPP11 crystal. The catalytic domain is indicated as CD_A and CD_B, and the helical domain is indicated as HD_A and HD_B in subunits A and B, respectively. **(c)** Close-up view of the active site depicting the catalytic serine in FpDPP11 as sticks. Serine substitution to alanine in PeDPP11 and PgDPP11 is also shown as sticks. The mutation does not alter the local secondary structure conformation.

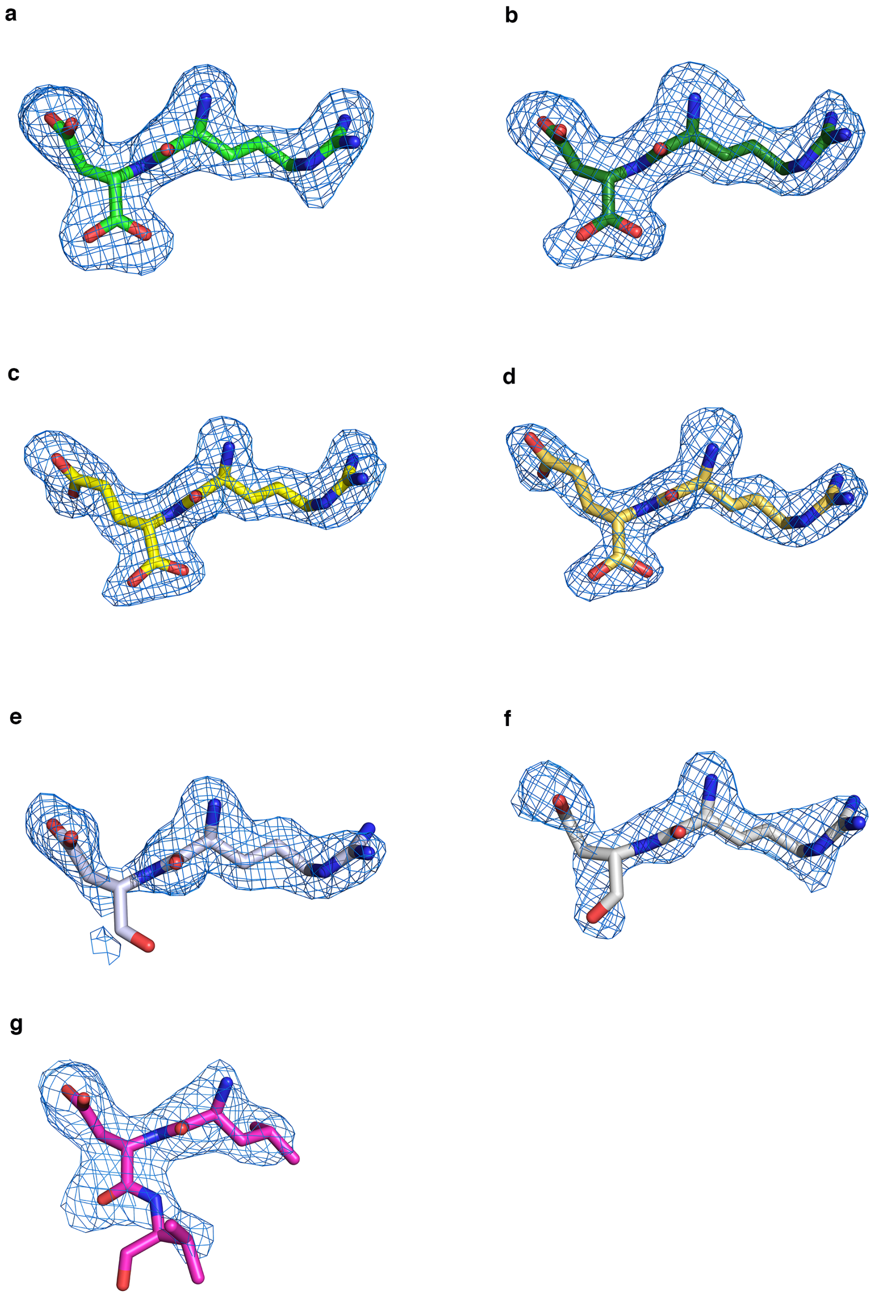

Supplementary Figure S7. Peptide omit maps contoured at 3σ. PeDPP11:RD chain D (**a**) and chain E (**b**). PeDPP11:RE chain D (**c**) and chain E (**d**). FpDPP11:RD chain F (**e**) and chain I (**f**). PeDPP11:LDVW **(g)**.

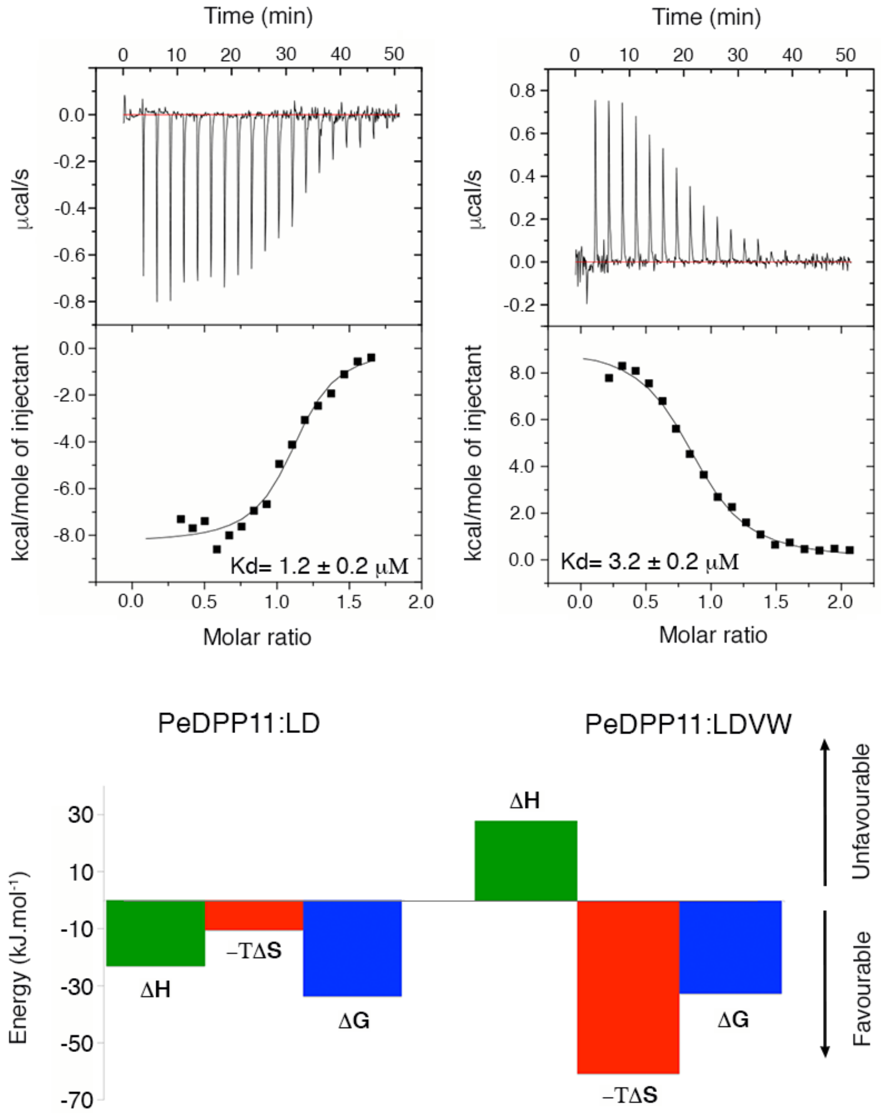

Supplementary Figure S8. Microcalorimetric analysis. Isothermal titration calorimetry experiments performed in phosphate buffer by titrating LD **(left panel)** and LDVW **(right panel)** into PeDPP11. Upper panel shows time-dependent deflection of heat for each injection (top). Integrated calorimetric data for the respective interactions (bottom). The continuous curve represents the best fit using a one-site binding model. Lower panel shows the graphical representation of thermodynamics parameters.

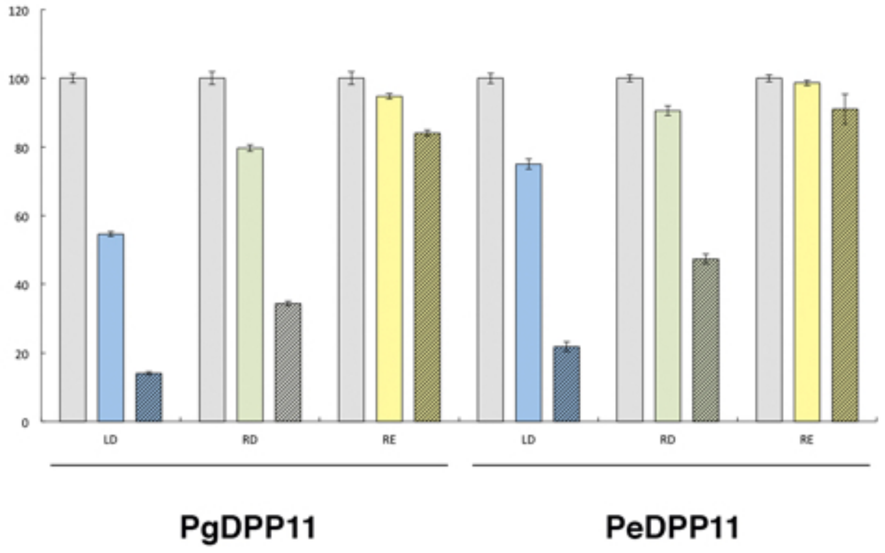

Bacteria	Substrate	IC50 (mM) LD	RD	RE
P. gingivalis	LD-MCA	0.056	0.086	1.48
P. endodontalis		0.059	0.172	58.0
P. gingivalis	LE-MCA	0.022	0.058	2.60
P. endodontalis		0.048	0.074	4.60

Supplementary Figure S9. Inhibitory effects of dipeptides on DPP11 activity. **Upper panel:** Peptidase activity was determined using 20 μM either LD- or LE-MCA as substrate in the absence (grey columns) or presence of dipeptides (LD, RD and RE coloured in blue, green and yellow, respectively) at 20 μM (open column) and 200 μM (hatched column). Values are means of three separate experiments. Error bars represent standard deviations (s.d.). **Lower panel:** The dipeptide concentration giving 50% inhibition [IC_{50} (mM)] was determined by intra- ($IC_{50} \leq 0.2$ mM) or extra-polation ($IC_{50} > 0.2$ mM) of inhibition data.

Supplementary Table S1a. Root mean square deviations (Å).

	Unbound PeDPP11	
	Helical domain	Catalytic domain
**PeDPP11:LDVW**	1.9 (248 out of 269 Cα atoms)	0.4 (354 out of 401 Cα atoms)
**PeDPP11:RD**	0.9 (227 out of 287 Cα atoms)	0.4 (347 out of 404 Cα atoms)
**PeDPP11:RE**	0.9 (231 out of 288 Cα atoms)	0.4 (345 out of 403 Cα atoms)
**PeDPP11$_{altconf}$**	2.3 (224 out of 257 Cα atoms)	0.4 (355 out of 399 Cα atoms)

Supplementary Table S1b. PeDPP11 domain B-factors (Å^2).

	**ChainA**		**ChainB**	
	**Helical domain**	**Catalytic domain**	**Helical domain**	**Catalytic domain**
**Unbound PeDPP11**	55.8	31.4	62.8	54.0
**PeDPP11:RD**	60.3	51.0	95.0	64.3
**PeDPP11:RE**	45.7	41.9	102.9	51.2
**PeDPP11:LDVW**	57.8	48.0		
**PeDPP11$_{altconf}$**	54.4	44.6		

Supplementary Table S2a. Amino acid residues forming the subsites of the peptide binding pocket in PeDPP11:LDVW.

Subsite	Residues
S2 (Leu)	His85, Asn217, Trp218, Arg222, Asp226, Asn332, Arg336, Phe668, Asp669
S1 (Asp)	His85, Trp218, Thr647, Thr648, Gly649, Gly650, Asn651, Ala652, Asn667, Phe668, Asp669, Arg670
S1' (Val)	Gly68, Cys69, His85, Asn332, Gly649, Gly650, Ala652, Phe668

Supplementary Table S2b. Amino acid residues forming the subsites of the peptide binding pocket in PeDPP11:RD.

Subsite	
S2 (Arg)	His85, Lys210, Ser213, Asn217, Trp218, Arg222, Asp226, Ala328, Gln331, Asn332, Arg336, Phe668, Asp669
S1 (Asp)	Gly68, Cys69, His85, Trp218, Thr647, Thr648, Gly649, Gly650, Asn651, Ala652, Asn667, Phe668, Asp669, Arg670

Supplementary Table S2c. Amino acid residues forming the subsites of the peptide binding pocket in PeDPP11:RE.

Subsite	
S2 (Arg)	His85, Ser213, Asn217, Trp218, Arg222, Asp226, Ala328, Ser329, Gln331, Asn332, Arg336, Phe668, Asp669
S1 (Glu)	Gly68, His85, Trp218, Thr647, Thr648, Gly649, Gly650, Asn651, Ala652, Asn667, Phe668, Asp669, Arg670
